# Agreement between QuantiFERON^®^-TB Gold In-Tube and the tuberculin skin test and predictors of positive test results in Warao Amerindian pediatric tuberculosis contacts

**DOI:** 10.1186/1471-2334-14-383

**Published:** 2014-07-11

**Authors:** Lilly M Verhagen, Mailis Maes, Julian A Villalba, Adriana d’Alessandro, Lazaro Perez Rodriguez, Mercedes F España, Peter WM Hermans, Jacobus H de Waard

**Affiliations:** 1 Laboratorio de Tuberculosis, Instituto de Biomedicina, Universidad Central de Venezuela, Caracas, Venezuela; 2 Department of Pediatrics, Laboratory of Pediatric Infectious Diseases, Radboud University Medical Centre, PO Box 9101, 6500 HB Nijmegen, The Netherlands; 3 Department of Medicine, University of Cambridge, Cambridge, UK; 4 Lovelace Respiratory Research Institute, Albuquerque, USA; 5 ASIC Elena Cotua Municipio Antonio Díaz Misión Medica Cubana Delta Amacuro Venezuela, Tucupita, Venezuela; 6 Programa Nacional Integrado de Control de la Tuberculosis, Ministerio de Salud, Caracas, Venezuela

**Keywords:** Tuberculosis, Indigenous children, Diagnostics, Child tuberculosis contacts

## Abstract

**Background:**

Interferon-gamma release assays have emerged as a more specific alternative to the tuberculin skin test (TST) for detection of tuberculosis (TB) infection, especially in Bacille Calmette-Guérin (BCG) vaccinated people. We determined the prevalence of *Mycobacterium tuberculosis* infection by TST and QuantiFERON^®^-TB Gold In-Tube (QFT-GIT) and assessed agreement between the two test methods and factors associated with positivity in either test in Warao Amerindian children in Venezuela. Furthermore, progression to active TB disease was evaluated for up to 12 months.

**Methods:**

163 HIV-negative childhood household contacts under 16 years of age were enrolled for TST, QFT-GIT and chest X-ray (CXR). Follow-up was performed at six and 12 months. Factors associated with TST and QFT-GIT positivity were studied using generalized estimation equations logistic regression models.

**Results:**

At baseline, the proportion of TST positive children was similar to the proportion of children with a positive QFT-GIT (47% vs. 42%, p = 0.12). Overall concordance between QFT-GIT and TST was substantial (kappa 0.76, 95% CI 0.46-1.06). Previous BCG vaccination was not associated with significantly increased positivity in either test (OR 0.68, 95% CI 0.32-1.5 for TST and OR 0.51, 95% CI 0.14-1.9 for QFT-GIT). Eleven children were diagnosed with active TB at baseline. QFT-GIT had a higher sensitivity for active TB (88%, 95% CI 47-98%) than TST (55%, 95% CI 24-83%) while specificities were similar (respectively 58% and 55%). Five initially asymptomatic childhood contacts progressed to active TB disease during follow-up.

**Conclusion:**

Replacement of TST by the QFT-GIT for detection of *M. tuberculosis* infection is not recommended in this resource-constrained setting as test results showed substantial concordance and TST positivity was not affected by previous BCG vaccination. The QFT-GIT had a higher sensitivity than the TST for the detection of TB disease. However, the value of the QFT-GIT as an adjunct in diagnosing TB disease is limited by a high variability in QFT-GIT results over time.

## Background

Tuberculosis (TB) in children is an important public health problem. Worldwide, at least half a million children become ill with TB and as many as 70,000 children die of TB each year [1]. A unique aspect of TB in young children is the rapid progression to disease, typically within the first year following infection, unlike in adults, where latent TB infection (LTBI) can persist for decades without progression into active disease [2]. The tuberculin skin test (TST) is a simple and relatively cheap test for the detection of LTBI. However, the specificity of the TST is affected by cross-reactivity with the Bacille Calmette-Guérin (BCG) vaccine and exposure to nontuberculous mycobacteria [3]. Interferon-gamma release assays (IGRAs) have emerged as a more specific alternative to the TST [4].

A systematic review of studies comparing IGRAs and TST in children concluded that the tests have similar accuracy for the detection of LTBI or active TB [5]. However, a meta-analysis of QuantiFERON^®^-TB Gold In-Tube (QFT-GIT) sensitivity in children and adults in high burden vs. low burden TB settings showed a clear trend towards lower QFT-GIT sensitivity in high burden settings [6].

In several adult studies, a relationship between CXR or chest computed tomography (CT) lesions indicative of previous TB and TST or IGRA positivity has been observed [7-10]. The observation that rates of IGRA positivity are higher in individuals with CXR and CT lesions suggesting old healed TB than in those without such lesions [8] is in contrast with the suggestion that IGRA results become negative once the mycobacteria enter a dormant state [11]. Studies performing serial IGRA testing in childhood household contacts are scarce and suggest that both conversions and reversions of IGRA results occur [12-14]. None of these studies assessed the relationship between IGRA or TST results and CXR lesions suggestive of previous TB.

Although IGRAs are more costly and technically complex than the TST, they offer the advantage of requiring only a single patient visit whereas the TST requires a health care professional reading the induration size at 48 to 72 hours. Published reports regarding return rates for TST assessment vary widely from 12% to 72% [15-19]. Low return rates are especially worrisome when the TST is used as an adjunct in diagnosing TB disease, since this can lead to under-diagnosis of TB if children that do not show up for a return visit remain undiagnosed.

In Venezuela, the national prevalence of TB is moderate (25–87 per 100,000 inhabitants) [20], but an extraordinarily high TB prevalence (3190 per 100,000) has been reported in Warao Amerindian children [21]. This indigenous population resides in the Orinoco River Delta, where they live in wooden houses raised on stilts along the Orinoco River banks. Tuberculin purified protein derivative used for TST testing is not widely available in the Warao communities, although surveys using TST testing have been performed [22]. The diagnosis of TB disease and the subsequent initiation of TB treatment occurs through the Orinoco Delta Department of the Venezuelan National TB Program situated in the hospital in Tucupita, the state capital city of the Orinoco Delta on the mainland. When an individual is diagnosed in this hospital, TB treatment is transported to the community in which the patient lives, where it is given to community nurses that are responsible for the delivery and supervision of TB treatment. Isoniazid preventive therapy is currently not routinely provided for childhood contacts of adult TB patients in the Orinoco Delta. While some Warao people have family members who live in Tucupita, many do not which means that they do not have a place to stay when visiting Tucupita to go the hospital. As a consequence, many Warao people prefer to return to their communities the same day rather than staying in Tucupita for the night. This leads to under-diagnosis and subsequent under-treatment of TB when TST results are awaited in order to make a definite TB diagnosis. The IGRA could potentially decrease the under-diagnosis related to failure to adhere to return visits in this specific setting.

The primary objectives of our study were 1) to determine the prevalence of *Mycobacterium tuberculosis* infection by TST and QFT-GIT and assess agreement between the two test methods and 2) to identify characteristics associated with TST positivity and QFT-GIT positivity in childhood TB contacts. Secondary objectives were the assessment of the QFT-GIT and TST performance to detect TB disease and predict the development of TB disease and their relationship with radiological features possibly indicating the presence of TB sequelae.

## Methods

### Study design and setting

Warao Amerindian children under 16 years of age residing in the municipalities Antonio Díaz and Pedernales in the Orinoco Delta in Northeastern Venezuela who were household contacts of culture-confirmed TB patients who registered for TB treatment in the Venezuelan National TB Control Program were included between May 2010 and December 2011. All included household contacts underwent QFT-GIT testing, TST and CXR examinations. A previously published cross-sectional analysis reported on the association of TST and QFT-GIT positivity with helminth infections and cytokine profiles in asymptomatic Warao childhood TB household contacts [23]. For the prospective study presented here, all childhood contacts of culture-confirmed TB patients, both symptomatic and asymptomatic, from 0–15 years of age who were identified within six months after registration of the index case between May 2010 and December 2011 were included, if they did not meet the exclusion criteria. Exclusion criteria were absence of a written informed consent, untracibility of the registered index patients and their contacts for inclusion, a positive Human Immunodeficiency Virus (HIV) test at any time point, TST administration in the three months before inclusion, laboratory documented anemia (hemoglobin <9 g/dl) at inclusion and previous TB treatment.

At six-month follow-up, CXR and QFT-GIT were taken of children that were diagnosed with TB and put on TB treatment at inclusion. In addition, active follow-up was performed in order to screen for children that did not have active TB at inclusion but developed active TB within the first six months after inclusion. To this end, a TST and physical examination were performed in all encountered children. Subsequently, those children that converted from TST negative to TST positive as well as those children presenting any sign or symptom that may be associated with active TB (ie, any of reported or documented fever, cough, weight loss or failure to thrive, lethargy or decrease in playfulness/activity or enlarged lymph nodes or auscultatory findings that may indicate airway disease) underwent CXR and QFT-GIT.

At 12-month follow-up, all included children were followed up with CXR and QFT-GIT, irrespective of TST or physical examination results. Repeat TST testing during follow-up was only performed in children that were previously TST negative. In addition to the active follow-up at six and 12 months, the cohort was monitored by passive follow-up with a focus on TB development based on reports to the Orinoco Delta Department of the National TB Control Program. Furthermore, four of the authors (LMV, MM, LPR and JAV) resided in the Orinoco Delta at the time of research where they periodically visited communities in the study region.

### Sample collection

Blood was collected for QFT-GIT assays [24] and Human Immunodeficiency Virus (HIV) antibody testing using the Determine™ (Abbot Laboratories, Illinois, USA) HIV 1/2 rapid test. The tubes for the QFT-GIT assay were incubated on site with a portable incubator. HIV testing was performed upon inclusion and at 12-month follow-up in all children. The TST was performed using 0.1 mL of tuberculin purified protein derivative (PPD RT23, Statens Seruminstitut, Copenhagen, Denmark). Reading was performed by trained professionals measuring the palpable transverse induration on the volar surface of the forearm between 48 and 72 hours after administration. Antero-posterior and lateral CXRs were taken. Two independent experts, blinded to all clinical information except for age, evaluated the CXRs. Where the two experts disagreed, a third expert was consulted and final consensus was achieved. A sputum sample was collected from all children who could expectorate with gastric aspirates taken from all children under 6 years of age upon inclusion. During follow-up, sputum or gastric aspirate samples were only taken from symptomatic children. Specimens were cultured on Middlebrook (7H9) liquid broth-based media and on Ogawa solid media.

### Definitions

Confirmed TB was defined as isolation of *M. tuberculosis* on culture. PCR-restriction analysis of the hsp65 gene (PRA) was performed to differentiate *M. tuberculosis* from nontuberculous mycobacteria. Probable TB was defined as clinical signs and symptoms of TB and radiographic findings consistent with intrathoracic TB as defined by Marais et al. [25], and either (1) a positive TST or QFT-GIT or (2) histopathologic findings compatible with TB, without positive mycobacterial culture results. Possible TB was defined as clinical signs and symptoms of TB and abnormal CXR findings not consistent with but possibly related to active TB (eg, nonspecific shadows) and either (1) or (2). In children <3 years of age, a diagnosis of probable or possible TB was also made when clinical signs and symptoms of TB were present together with radiographic findings but without a positive TST or QFT-GIT, because in this age group these T-cell based tests have low sensitivity for TB disease [26]. When clinically indicated, additional examinations to diagnose extrapulmonary forms of TB were performed.

CXRs were classified as (1) normal (no abnormalities suggestive of current or past TB observed), (2) active TB or (3) radiographic lesions possibly related to past pulmonary TB (TB sequelae). The latter category was defined as the presence of at least one of the three radiological features (calcification, parenchymal destruction with fibrosis, bronchiectasis) defined as ‘consequences of previous pulmonary tuberculosis’ by Marais et al. [25]. CXR findings regarded as compatible with bronchiectasis were saccular changes or cylindrical outlines of airways that widened as airways extended into the lung periphery [27,28].

Previous BCG vaccination was defined as the presence of a BCG scar. Height and weight were recorded and transformed into weight-for-height, height-for-age, and body mass index (BMI)-for-age Z scores based on WHO standard reference populations [29,30] using WHO anthro software [31]. Children under 5 years of age with weight-for-height or height-for-age Z scores <−2 standard deviations (SD) were defined as malnourished. Children aged 5 to 15 years with BMI-for-age or height-for-age Z scores <−2 SD were defined as malnourished [32-34].

Venezuelan National TB Program guidelines regard a TST ≥10 mm 48–72 hours after injection as positive. A TST conversion was defined as having a negative TST at baseline and a TST ≥10 mm at any follow-up time point. An induration of <10 mm was considered TST negative.

A positive QFT-GIT was defined as a TB antigen minus nil (negative control) value ≥0.35 IU/ml and ≥25% of nil value. A nil value of >8.0 IU/ml or a mitogen minus nil of <0.5 IU/ml was classified as indeterminate. QFT-GIT conversion or reversion was defined as respectively a negative to positive or a positive to negative change according to manufacturer’s criteria (ie, baseline IFN-γ <0.35 IU/ml and follow-up IFN-γ ≥0.35 IU/ml or baseline IFN-γ ≥0.35 IU/ml and follow-up IFN-γ <0.35 IU/ml respectively) [24].

### Ethical considerations

The nature and objectives of the study were explained to the parents of exposed children in Spanish and/or in their native language. The study was approved by the ethical committee of the Instituto de Biomedicina, the Regional Health Services, and the Delta Amacuro Indigenous Health Office (Servicio de Atención y Orientación al Indígena). Children were enrolled if their parents or primary caregivers provided written informed consent. If parents or primary caregivers were illiterate, consent forms were read to them in Spanish and/or in their native language by Spanish-Warao bilingual native interpreters and signed by means of a thumb print.

Children diagnosed with confirmed, probable or possible TB were treated with a standard six month anti-TB regime as recommended by the Venezuelan National TB Control Program.

### Sensitivity, specificity, positive and negative predictive value calculations

The sensitivity and specificity of the TST and QFT-GIT for active TB were calculated. For these estimations, children that died from an unknown cause as well as children diagnosed with TB at six or 12 month follow-up were excluded. Additionally, the positive and negative predictive values of TST and QFT-GIT testing for the identification of initially asymptomatic children that progressed to active TB and would thus have benefited from preventive treatment were calculated.

### Statistical analysis

Test concordance was calculated using the Kappa (ĸ) coefficient statistic. Categorical unpaired and paired data were compared using respectively Chi-square (or Fisher’s exact test, as appropriate) and McNemar’s tests. For unpaired continuous variables, the unpaired Student’s t test or the nonparametric Mann-Whitney’s test was used depending on whether or not the variables were normally distributed (Kolmogorov-Smirnov’s test, p > 0.05).

Generalized estimation equations (GEEs) were used to fit multivariable logistic regression models aimed at identifying possible associations between QFT-GIT or TST positivity (dependent variable) and BCG vaccination, age, sex, malnourishment and duration of exposure (independent variables) and between the presence of possible TB sequelae on CXR (dependent variable) and IFN-γ level, TST induration, BCG vaccination, age, sex, malnourishment and duration of exposure (independent variables). GEEs account for correlation and lack of independence of responses for contacts with an index TB case in common (clusters within households). Results were reported as adjusted odds ratios (OR) with 95% confidence intervals (CI). SPSS software for windows, version 20.0 (SPSS Inc, Chicago, IL, USA) was used for statistical analyses. Statistical significance was set to p-value <0.05.

## Results

**Figure 1:**
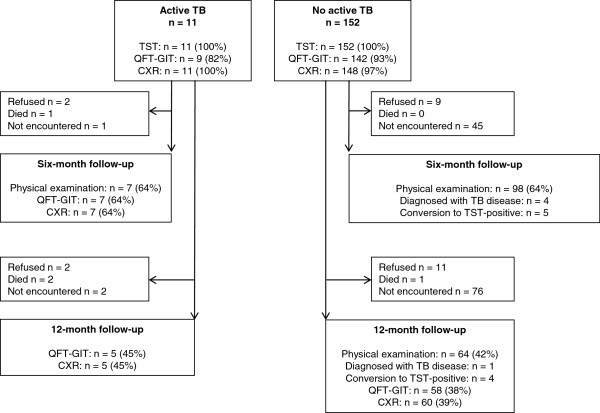
Study recruitment profile and number of children assessed at different time points.

**Figure 2:**
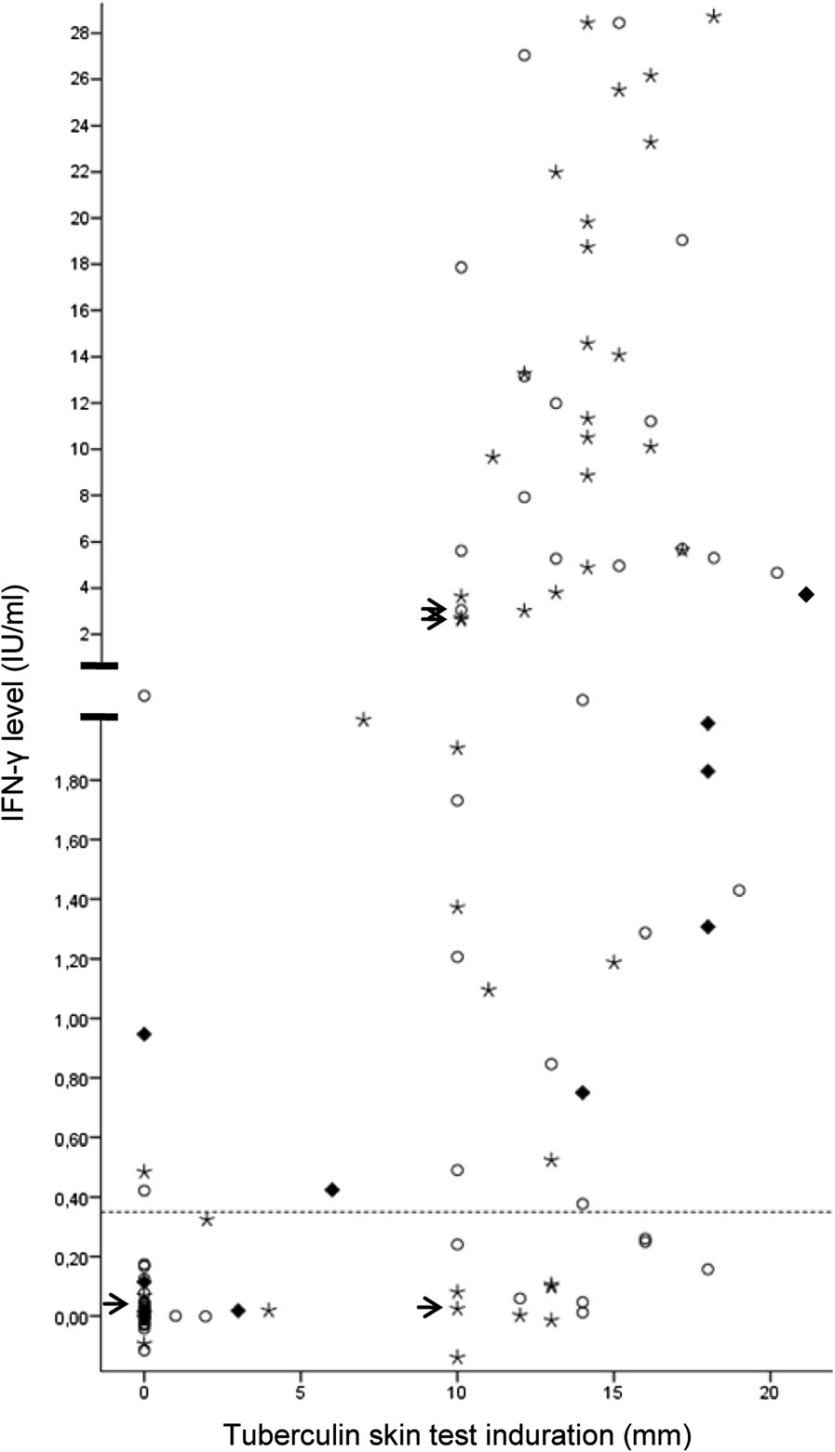
**QuantiFERON**^**®**^**-TB Gold in-tube assay (QFT-GIT) and tuberculin skin test (TST) data upon inclusion from the 149 included children with both results and chest X-rays (CXRs) available.** The QFT response is the level of IFN-γ (IU/ml) in the tuberculosis (TB) antigen-stimulated plasma sample with that for the negative control subtracted. Results for those 9 children who were diagnosed with TB at inclusion are represented by *solid diamonds* (in 2 children diagnosed with TB no QFT-GIT was performed). Results for those who showed TB sequelae at CXR are represented by *asterisks*. The arrows point to the results of 4/5 children (in 1 child no QFT-GIT was performed at inclusion) who progressed to active TB during follow-up. The *dotted line* represents the 0.35 IU/ml cutoff for the QFT-GIT test.

At baseline 163 household contacts were enrolled for TST testing, QFT-GIT and CXR (Figure 1). Six registered adult TB patients were untraceable and in one household, parents were not willing to enroll their children in the study. For 149 children (91%), results for TST and QFT-GIT as well as CXR were available (Figure 2). The mean age of included children was 7.7 years (standard deviation 4.2 years). Additional data at inclusion, classified by age group, are summarized in Table 1.

**Table 1:** Characteristics of study population classified by age group

**Characteristics, n (%)**	**0-5 years**	**6-10 years**	**11-15 years**
	**n = 54 (33%)**	**n = 62 (38%)**	**n = 47 (29%)**
Male	29 (54)	38 (61)	25 (53)
Female	25 (46)	24 (39)	22 (47)
TST positive	6 (11)	32 (52)	36 (77)
Active TB disease	2 (4)	5 (8)	4 (9)
Malnourished	31 (57)	20 (32)	13 (28)
BCG vaccinated	41 (76)	58 (94)	41 (87)

Of the 163 children, 11 (7%, 95% CI 3-12%) were diagnosed with active TB at baseline. In the children with both a TST and a QFT-GIT result available, the proportion of children with a positive TST was somewhat but not significantly higher than the proportion of children with a positive QFT-GIT (47% vs. 42%, p = 0.12). When excluding indeterminate results, the concordance between TST and QFT-GIT was substantial (ĸ 0.76, 95% CI 0.46-1.06, Table 2). The concordance between TST and QFT-GIT was lower but still substantial in BCG vaccinated children compared with children who were not BCG vaccinated (ĸ 0.74, 95% CI 0.62-0.86 vs. ĸ 0.89, 95% CI 0.68-1.09 respectively). Previous BCG vaccination, malnourishment and duration of exposure were not significantly associated with either TST or QFT-GIT positivity in multivariable analysis (Table 3). TST positivity rates were higher in males compared with females (OR 3.7, 95% CI 1.5-9.4) and increased with each year of age (OR 1.4, 95% CI 1.2-1.7). QFT-GIT positivity was not significantly associated with these factors in multivariable analysis (Table 3).

**Table 2:** **Concordance rate of tuberculin skin test and QuantiFERON**^**®**^**-TB Gold In-Tube at inclusion**

	**Tuberculin skin test, n (%)**		**Total, n**	**QuantiFERON**^**®**^**-TB Gold In-Tube, n (%)**			**Total, n**	**Kappa (95% CI)**
	**Positive**	**Negative**		**Positive**	**Negative**	Indeterminate*		
**Total**	74 (45)	89 (55)	163	63 (42)	77 (51)	11 (7)	151	0.76 (0.46-1.06)
**Active TB**	6 (55)	5 (45)	11	7 (78)	1 (11)	1 (11)	9	0.39 (−0.19-0.97)
**Chest-Xray in asymptomatic children**								
TB sequelae	34 (56)	27 (44)	61	30 (52)	24 (41)	4 (7)	58	0.73 (0.54-0.91)
No features of TB	32 (37)	55 (63)	87	25 (31)	51 (62)	6 (7)	82	0.77 (0.62-0.92)
**BCG vaccination status**								
BCG scar present	65 (46)	75 (54)	140	55 (42)	67 (51)	9 (7)	131	0.74 (0.62-0.86)
BCG scar absent	9 (39)	14 (61)	23	8 (40)	10 (50)	2 (10)	20	0.89 (0.68-1.09)
**Age**								
0 - 4 years	4 (9)	41 (91)	45	2 (5)	32 (82)	5 (13)	39	0.79 (0.38-1.20)
5 - 15 years	70 (59)	48 (41)	118	61 (55)	45 (40)	6 (5)	112	0.69 (0.55-0.83)

*For calculation of kappa values, children with indeterminate QuantiFERON^®^-TB Gold In-Tube results were excluded.

**Table 3:** **Factors predicting tuberculin skin test (TST) and QuantiFERON**^**®**^**-TB Gold In-Tube (QFT-GIT) positivity in Warao Amerindian childhood contacts**

**Characteristics**	**QFT-GIT positive vs. QFT-GIT negative**	**TST positive vs. TST negative**
	**Odds ratio (95% CI)**	**Odds ratio (95% CI)**
**Age, years**	0.99 (0.84-1.2)	1.4 (1.2-1.7)
**Sex, male vs. female**	0.6 (0.13-3.2)	3.7 (1.5-9.4)
**Malnourished**	4.6 (0.93-22.2)	0.92 (0.50-1.7)
**BCG vaccination**	0.51 (0.14-1.9)	0.68 (0.32-1.5)
**Duration of exposure, ≥12 vs. <12 h/day**	1.4 (0.35-5.65)	0.81 (0.34-1.9)

### TST and QFT-GIT results in children with active TB at inclusion

At inclusion, 11 children were diagnosed with active TB, of which two were confirmed pulmonary TB, four were probable and four were possible intrathoracic TB. One child was diagnosed with intrathoracic and hip bone TB. Five children were TST negative and six had a positive TST result, corresponding to 55% sensitivity (95% CI 24-83%) and 55% specificity (95% CI 47-64%) of the TST for active TB. The QFT-GIT was not performed in two children with active TB due to logistical difficulties. Excluding these children as well as the children with an indeterminate QFT-GIT result, the QFT-GIT had a 88% sensitivity (95% CI 47-98%) and 58% specificity (95% CI 49-67%) for the diagnosis of active TB. However, the small sample size and the low number of children with active TB as well as the fact that TST or QFT-GIT positivity was one of the criteria for the diagnosis of active TB preclude firm conclusions regarding the value of QFT-GIT and TST testing for the diagnosis of active TB in this population.

### Progression to active TB during follow-up

At six-month follow-up, 105 children were clinically examined: 7 children that were diagnosed with TB at inclusion and 98 children that were asymptomatic at inclusion (Figure 1). Follow-up examinations confirmed a diagnosis of possible or probable TB in four of these 98 children (4%). At 12-month follow-up, 64 initially asymptomatic children were encountered (Figure 1) and TB was diagnosed in one additional boy that did not present with TB disease at inclusion or six-month follow-up. The mean age of the five children that developed active pulmonary TB during follow-up was lower than the mean age of all included children (respectively 4 vs. 8 years). Demographic characteristics and test results of these children are summarized in Table 4. None of these five children converted from either TST negative to positive or from QFT-GIT negative to positive. Three of the five children were TST positive at inclusion and two also had a positive QFT-GIT result (Table 4, Figure 2). To estimate the value of TST and QFT-GIT testing for the identification of these children that would benefit from preventive treatment, we calculated their positive and negative predictive values. Of the 76 initially asymptomatic QFT-GIT negative contacts, two developed active TB, yielding a negative predictive value of 97% (95% CI 91-100%). Of the 84 initially asymptomatic TST negative children, two developed TB, resulting in a negative predictive value of 98% (95% CI 92-100%). Positive predictive values were much lower, 4% (95% CI 1-12%) and 3% (95% CI 0-10%) for QFT-GIT and TST respectively.

**Table 4:** Demographics and test results for the five contacts who progressed to active tuberculosis during follow-up

**Age, yr**	**Sex**	**BCG**	Malnourished*	**Duration contact (h/day)**	**TB diagnosis**	**Time point diagnosis**	**QFT-GIT**				**TST**				**Chest X-ray**	
							**Inclusion**		**TB diagnosis**		**Inclusion**		**TB diagnosis**		**Inclusion**	**TB diagnosis**
							**Result**	**IU/ml**	**Result**	**IU/ml**	**Result**	**mm**	**Result**	**mm**		
0 (6 mo)	F	Yes	Yes	<12	Probable	6 mo	Nd		Ind	−23.123	Neg	0	Neg	2	Segmental calcifications	Segmental calcifications, Ghon focus with cavitation, lymph node disease with tracheal compression
1	M	Yes	Yes	<12	Possible	6 mo	Neg	0.024	Neg	0.012	Pos	10	Nd		Segmental parenchymal destruction paracardial region right lung	Segmental parenchymal destruction paracardial region right lung, bilateral parenchymal infiltrates lower lobes
1	M	Yes	Yes	≥12	Probable	12 mo	Neg	0.012	Neg	0.000	Neg	0	Neg	0	No abnormalities	Alveolar consolidation right upper lobe
6	M	Yes	No	<12	Possible	6 mo	Pos	3.045	Neg	0.133	Pos	10	Nd		No abnormalities	Lobar alveolar consolidation right middle lobe
11	M	Yes	No	<12	Probable	6 mo	Pos	2.701	Pos	3.394	Pos	10	Nd		Segmental calcifications	Segmental calcifications, right hilar lymph node enlargement, alveolar consolidation left upper lobe

*yr* = years, *mo* = months, *h* = hours, *F* = Female, *M* = Male, *BCG* = Bacille Calmette - Guérin, *TB* = Tuberculosis, *QFT-GIT* = QuantiFERON^®^-TB Gold In-Tube assay, *TST* = Tuberculin skin test, *Nd* = Not done, *Ind* = Indeterminate, *Pos* = Positive, *Neg* = Negative.

*Reflects nutritional status upon inclusion.

### TST and QFT-GIT results during follow-up

At inclusion, 89 children were TST negative (Table 2). In 60 (67%) of these initially TST negative children, a repeat TST was performed during follow-up. There were no differences in the age or sex characteristics of those initially TST negative contacts in which a repeat TST was performed vs. those without a follow-up TST. Nine children (15% of all children with a repeat TST result) converted from TST negative to TST positive during follow-up.

QFT-GIT results at both inclusion and 12 month follow-up were available for 57 children. There was no significant age difference between these children and the (163–57) 106 children for whom QFT-GIT results at both time points were not available. Girls were more likely to have follow-up QFT-GIT results than boys (44% vs. 28%, p = 0.041). One third of the 57 children with both results available (n = 19) had a QFT-GIT result at 12-month follow-up that was not identical to their result at inclusion (Additional file 1: Table S1). There were no significant differences in the age and sex characteristics of children that changed their QFT-GIT result compared with those in which the follow-up QFT-GIT result was identical to the recruitment result. Of the initially QFT-GIT positive children with valid follow-up data, 62% reverted to negative or indeterminate while only 15% of the initially QFT-GIT negative children showed a different result at 12-month follow-up (p < 0.01, Additional file 1: Table S1). Median quantitative recruitment QFT-GIT responses in QFT-GIT positive contacts that reverted to negative were lower than responses in those QFT-GIT positive contacts that remained positive (1.3 IU/ml, range 0.85-8.8 vs. 3.7 IU/ml, range 1.2-5.3). Similarly, median quantitative recruitment responses in children that converted from QFT-GIT negative to positive were somewhat higher than responses in those QFT-GIT negative contacts that remained negative (0.021 IU/ml, range −0.001 to 0.32 vs. 0.002 IU/ml, range −7.6 to 0.17).

### Chest X-ray findings in children without active TB

In 61/148 (41%) children without active TB and valid CXR results at inclusion at least one radiographic feature of possible TB sequelae was detected. Most children (n = 46, 31%) showed calcifications (Table 5). In 39 (85%) of the children with calcifications, this was the sole abnormality detected. A total number of 17 children (11%) showed radiological features suggestive of bronchiectasis (Table 5) and in 11 of them (65%) this was the sole abnormality detected.

**Table 5:** Children without active TB who had radiographic lesions possibly associated with tuberculosis sequelae at inclusion

Radiographic characteristic*	Number of children, n (%)**
**Calcification**	46 (31)
Segmental	17 (11)
Lobar	2 (1)
Unilateral	3 (2)
Bilateral	24 (16)
**Parenchymal destruction with fibrosis**	5 (3)
Segmental	4 (3)
Lobar	0 (0)
Unilateral	0 (0)
Bilateral	1 (0)
**Bronchiectasis**	17 (11)
Segmental	2 (1)
Lobar	3 (2)
Unilateral	2 (1)
Bilateral	10 (7)

*The herementioned radiographic characteristics were defined as “consequences of previous pulmonary tuberculosis” in the disease classification proposed by Marais et al. [25].

**Percentages reflect the proportion of the total number of children without active TB of whom a CXR was taken (n = 148).

Of the 148 children, 57 (39%) had a repeat CXR at 12-month follow-up. Of the 57 children with valid CXR results at both inclusion and 12-month follow-up, six (11%) showed TB sequelae at the follow-up CXR while these were not present at the CXR taken upon inclusion. All six children showed calcifications (bilateral in four children and segmental in two children); no child showed parenchymal destruction or bronchiectasis at 12 month follow-up that was not present upon inclusion. Five of the six children were QFT-GIT negative at inclusion; in one child no QFT-GIT was performed. At 12 month follow-up, the appearance of calcifications was not accompanied by a reversion of the QFT-GIT results in these five children; all QFT-GIT levels remained around 0. In one child the appearance of segmental calcifications on CXR was accompanied by a conversion from TST negative (0 mm) to TST positive (13 mm) while the QFT-GIT level did not change (respectively −0.031 and 0.007 IU/ml).

When the presence of possible TB sequelae on CXR was taken as a dependent variable in multivariable logistical GEE regression analysis, four independent factors were found to be significantly associated: IFN-γ level in IU/ml, age, sex and BCG vaccination status (Table 6). Every unit increase of IFN-γ per milliliter was marginally significantly associated with an increased risk of having TB sequelae (p = 0.049). Furthermore, the risk of having TB sequelae increased significantly with each year of age (OR 1.3, 95% CI 1.0-1.6, p = 0.029), was higher in boys than in girls (OR 1.8, 95% CI 1.1-3.0) and in BCG vaccinated children compared with unvaccinated children (OR 2.7, 95% CI 1.2-6.5). Additionally, there was a trend towards a lower risk of having TB sequelae on CXR in malnourished children (p = 0.055) in multivariable analysis. TST induration diameter was not found to be associated with the presence of possible TB sequelae (p = 0.44, Table 6).

**Table 6:** Results of multivariable logistic GEE regression analysis for the presence of TB sequelae on chest X-rays vs. normal chest X-rays at inclusion*

**TB sequelae on CXR**	**Odds ratio**	**95% CI**	**p-value**
IFN-γ level, IU/ml**	1.05	1.0-1.1	0.049
**TST induration diameter, mm**	0.95	0.84-1.08	0.44
**Age, years**	1.3	1.0-1.6	0.029
**Sex, male vs. female**	1.8	1.0-3.0	0.034
**Malnourished**	0.39	0.15-1.0	0.055
**BCG vaccination**	2.7	1.2-6.4	0.020
**Duration of exposure, ≥12 h/day vs. <12 h/day**	0.90	0.55-1.5	0.69

*Individuals who had active TB, indeterminate QFT-GIT results and those in whom a CXR and/or QFT-GIT was not performed (n = 33) were excluded from this analysis. A total of 55/130 remaining household contacts had radiological features of TB sequelae. Of these, 34 had calcifications as the lone abnormality.

**The level of IFN-γ (IU/ml) in the QFT TB antigen-stimulated plasma sample with that for the negative control subtracted.

## Discussion

To our knowledge, this study is the first to investigate the relative utility of QFT-GIT and TST in South American indigenous children. A substantial concordance between TST and QFT-GIT results was observed and BCG vaccination was not associated with significantly increased positivity in either test. Both tests had a high negative predictive value (≥97%) but a low positive predictive value (≤4%) for the identification of contacts that developed active TB disease over time. Two earlier prospective studies including child TB contacts in Japan [35] and Germany [36] also reported high negative predictive values and low positive predictive values of QFT assays for progression to active TB disease. However, the proportion of children that were lost to follow-up in our study was very high (Figure 1) compared with previous studies [35,36] and estimations and comparisons of positive and negative predictive values should thus be interpreted with caution.

Five children developed active TB during 12 month follow-up. This highlights the importance of the provision of preventive therapy to child contacts in this area. Screening of child contacts is not routinely carried out in the Orinoco Delta. Lack of staff expertise, the need for a minimum of two appointments to complete screening, transport costs and high healthcare worker workload have been identified as barriers to contact screening in rural Africa [37]. In rural South Africa, screening by five simple questions was sufficient to exclude TB disease in child contacts with a negative predictive value between 96% and 100% [38]. As community health workers are present in many Warao villages, educating them to use symptom-based approaches could potentially make screening of child TB contacts feasible. Supervision of preventive treatment by community health workers would be vital as poor adherence to unsupervised chemotherapy has been reported [39-41].

We did not observe a significant association of previous BCG vaccination with either TST or QFT-GIT positivity. Other studies performed in children from high burden areas also did not report an association between BCG vaccination and TST positivity [42-46]. In contrast, two studies including children from low burden regions did observe a significant association of BCG vaccination with TST positivity and/or a low agreement between TST and QFT-GIT results in BCG vaccinated children [36,47]. A review by Dheda et al. assessing IGRA and TST utility in high burden vs. low burden settings concluded that agreement between IGRAs and the TST depends on the BCG vaccination status of individuals in low burden countries, whereas the concordance of IGRAs with TST is modest to good and not clearly associated with BCG vaccination status in high burden settings [6].

We observed both QFT-GIT conversions and reversions among childhood contacts. Children with a recruitment positive QFT-GIT result were more likely to have a different result at 12 month follow-up than those with an initially negative QFT-GIT. Other longitudinal studies including childhood contacts in India and South Africa also reported QFT-GIT conversions and reversions. In contrast to our observations, these studies reported somewhat higher rates of follow-up QFT-GIT results differing from initial results in children who were initially QFT-GIT negative compared with initially QFT-GIT positive children [12,13]. The high number of children reverting from IGRA positive to negative in our study could merely be due to biological variations among IGRA positive individuals. An alternative explanation is proposed by Hill and colleagues, who suggest that IGRA responses are inherently transient and require continued exposure to TB antigens to maintain high frequencies. They argue that reversions reflect the life cycle of *M. tuberculosis*, where the mycobacterium enters a dormant state in which it may not reliably secrete ESAT-6 and CFP-10 [48]. However, our observation that the QFT-GIT response in IU/ml correlated significantly with CXR lesions suggestive of previous TB, does not support the theory that QFT-GIT responses decrease when clearing of TB infection occurs. Large comprehensive studies are needed to address this issue.

The percentage of Warao children that showed a different QFT-GIT result at follow-up compared with their recruitment result (33%) was higher than the overall rate of changing results in studies from India (14%) [12] and South Africa (28%) [13]. A Ugandan study showed a similar rate of changing results in five-year-old household contacts (30%) at follow-up only three weeks after inclusion. The authors conclude that the IGRA cannot be used alone (without TST) to diagnose TB infection [49]. Indeed, the observation that QFT-GIT results in our population show a high variability over time complicates the potential use of a single QFT-GIT test as a supportive test in the diagnosis of childhood TB. As long as it is not clear whether conversions and reversions correlate with the degree of clearing the TB infection or whether changes simply reflect instability of IGRAs, single test results should be interpreted with caution.

In a high number of children (41%) without active TB at least one radiographic feature of possible TB sequelae was observed on the CXR taken upon inclusion. In indigenous TB contacts 0–19 years of age residing in the Brazilian Amazon, a similar prevalence (33%) of radiographic features possibly associated with TB sequelae was observed and calcifications were also the most frequently observed abnormality [50]. Both in the Brazilian as well as in our study population reported TB rates are extraordinarily high (>30 times higher than national estimates) [21,51]. Possibly, the high number of children demonstrating TB sequelae on CXR is related to repeated TB exposure since early childhood. However, the CXR lacks specificity for detection of TB sequelae. Radiologic lesions suggestive of inactive TB are also noticed in for example histoplasmosis, a granulomatous disease caused by *Histoplasma capsulatum* that is endemic in Venezuela [52]. Further research with a longer follow-up is needed to identify the specific causes and prognosis of radiologic lesions.

We observed a significant association of the presence of possible TB sequelae on CXR with age, sex and BCG vaccination status. Young children with less mature immune responses may show less immune-mediated lung tissue destruction than older children. In mice infected with *M. tuberculosis*, BCG vaccination promoted pathological inflammation and lung lesions and this was mediated by interleukin (IL)-17 [53]. According to a recently published systematic review, BCG vaccination in humans produces dramatically high levels of IL-17 [54]. We speculate that repeated *M. tuberculosis* exposure in children living in high burden areas leads to more immune-mediated lung damage in those children that already developed an immune response following BCG vaccination compared with unvaccinated children.

A considerable number of children (n = 17, 11%) had CXR findings indicating bronchiectasis. Our CXR definition of bronchiectasis was previously used in Alaska native and Turkish children [27,28]. However, a CXR can suggest but not confirm the presence of bronchiectasis [55]. CT of the chest, especially high-resolution CT (HRCT), has become the “gold standard” imaging modality for diagnosing bronchiectasis [56]. (HR)CT equipment is unfortunately not available in the hospitals and health posts in the Warao communities. In indigenous children <15 years of age from remote communities in Australia the prevalence rate of HRCT-confirmed bronchiectasis was estimated to be 14.7/1000 [57]. There are many other pathologies that can contribute to bronchiectasis development apart from TB, eg, pertussis, influenza, congenital malformations or foreign body aspiration [58]. In Alaska native children and adults, only 4% of patients with probable (defined by clinical and CXR findings) and definite (diagnosed by bronchogram or CT) bronchiectasis had a history of pulmonary TB. The majority (74%) of patients included in the Alaskan study was diagnosed with asthma [28]. In a recently published cross-sectional survey a high prevalence of asthma symptoms (26%) in Warao Amerindian children aged between 2 and 10 years was observed [59]. The finding that most of the children with X-ray findings suggestive of bronchiectasis showed bilateral lesions (24/46, 52%) suggests that other pathologies than TB have contributed to the bronchiectatic lesions. In Canadian patients with bronchiectasis, previous TB was more often associated with unilateral than with bilateral lesions (58% vs. 42% respectively) [60]. Studies including a control group of children without a TB contact would be able to distinguish TB-related CXR lesions from CXR findings related to other pathologies. However, in a high burden setting where under-diagnosis and under-registration of cases occur it is difficult to identify such a comparison group.

A major limitation of our study was the limited number of children with valid active follow-up data. The low proportion of TST negative children in which repeat testing was performed and the low proportion of children with a valid QFT-GIT result at inclusion in which a follow-up QFT-GIT was performed (respectively 67% and 38%) preclude firm conclusions regarding TST conversions and QFT-GIT conversions and reversions. Notably, we cannot exclude the possibility that TST and QFT-GIT conversions were related to ongoing transmission within the community. Warao people are semi-nomadic and many migrate each year temporarily from their villages for agricultural purposes. Although passive follow-up was performed to screen for additional active TB cases, we cannot exclude the possibility that TB cases might have been missed. Additionally, children with a positive TST result at baseline did not receive a follow-up TST, and therefore we could not explore the occurrence of TST reversions. Study subjects with a BCG scar were reported as BCG vaccinated and vaccination cards were not examined. This may have led to under-reporting of the number of children with BCG vaccination since some BCG vaccinated children may not develop a scar. However, household-retained vaccination cards have also been identified as an insufficient source of information for estimating vaccination coverage in other areas [61]. Finally, although we included all child TB contacts of registered TB patients that were encountered and willing to participate, we cannot rule out the possibility that child TB contacts were missed due to under-registration of TB cases in the Orinoco Delta Department of the Venezuelan National TB Control Program.

## Conclusions

In Warao Amerindian childhood TB contacts, TST results were not significantly influenced by previous BCG vaccination and a substantial concordance between QFT-GIT and TST was observed in both BCG vaccinated and unvaccinated children. Replacement of TST by the QFT-GIT for estimation of LTBI prevalence rates is therefore not recommended in this resource-constrained setting. The QFT-GIT had a higher sensitivity than the TST in children with active TB. However, the potential use of a single QFT-GIT test as a supportive test in the diagnosis of childhood TB is limited by the high variability in QFT-GIT results over time. The relationship of radiographic features of possible TB sequelae with immune mechanisms and future progression to TB disease warrants further investigation.

## Competing interests

The authors declare that they have no competing interests.

## Authors’ contributions

LMV and MM participated in the design of the study, the collection of data, the statistical analysis and the interpretation of data and drafting the manuscript. JAV and LPR participated in the collection of data and helped to draft the manuscript. AA and MFE participated in the analyses of the collected data. PWMH participated in the design of the study and revised the manuscript critically for important intellectual content. JHW participated in the design of the study, coordinated the field work, advised on patient recruitment and revised the manuscript critically for important intellectual content. All authors read and approved the final manuscript.

## Pre-publication history

The pre-publication history for this paper can be accessed here:

http://www.biomedcentral.com/1471-2334/14/383/prepub

## Supplementary Material

Additional file 1: Table S1 QuantiFERON^®^-TB Gold In-Tube (QFT-GIT) results in the 57 children in whom a QFT-GIT at inclusion and at 12 month follow-up was performed. Click here for file
