# Supplementary material for: Agreement between QuantiFERON®-TB Gold In-Tube and the tuberculin skin test and predictors of positive test results in Warao Amerindian pediatric tuberculosis contacts
Source: BMC Infect Dis. 2014 Jul 11;14:383. doi: 10.1186/1471-2334-14-383 (PMC4227090; doi:10.1186/1471-2334-14-383)
Supplement: Additional file 1: Table S1 — QuantiFERON®-TB Gold In-Tube (QFT-GIT) results in the 57 children in whom a QFT-GIT at inclusion and at 12 month follow-up was performed. [file 1471-2334-14-383-S1.pdf]

Additional file 1: Table S1. QuantiFERON®-TB Gold In-Tube (QFT-GIT) results in the 57 children in whom a QFT-GIT at inclusion and at 12 month follow-up was performed

QFT-GIT result	Number of children, n (%)
Follow-up results of children with QFT-GIT negative inclusion result	39
Remained negative	33 (85)
Became positive	4 (10)
Became indeterminate	2 (5)
Follow-up results of children with QFT-GIT positive inclusion result	13
Remained positive	5 (38)
Became negative	5 (38)
Became indeterminate	3 (24)
Follow-up results of children with QFT-GIT indeterminate inclusion result	5
Remained indeterminate	0 (0)
Became negative	5 (100)
Became positive	0 (0)
